# The importance of temporal scale in distribution modeling of migratory Caspian Kutum, *Rutilus frisii*

**DOI:** 10.1002/ece3.70259

**Published:** 2024-09-23

**Authors:** Fateh Moëzzi, Hadi Poorbagher, Soheil Eagderi, Jahangir Feghhi, Carsten F. Dormann, Sabah Khorshidi Nergi, Kaveh Amiri

**Affiliations:** ^1^ Department of Fisheries, Faculty of Natural Resources University of Tehran Karaj Iran; ^2^ Department of Forestry and Forest Economics, Faculty of Natural Resources University of Tehran Karaj Iran; ^3^ Department of Biometry and Environmental System Analysis University of Freiburg Freiburg Germany; ^4^ Fishery Statistics and Economics Group Iranian Fisheries Organization (IFO) Tehran Iran; ^5^ Fishery Administration Karaj Alborz Province Iran

**Keywords:** boosted regression trees, Caspian Sea, data resolution, distribution modeling, fishing zones, *Rutilus frisii*, spatiotemporal dynamics

## Abstract

The choice of temporal resolution has high importance in ecological modeling, which can greatly affect the identification of the main drivers of an organism's distribution, considering the spatiotemporal dynamism of environmental predictors as well as organisms’ abundance. The present study aimed to identify the spatiotemporal distribution patterns of Caspian Kutum, *Rutilus frisii*, along the southern coast of the Caspian Sea, north of Iran, evaluating multiple temporal resolutions of data. The boosted regression trees (BRT) method was used to model fish catch distribution using a set of environmental predictors. Three temporal scales of data, including seasonal, sub‐seasonal, and monthly time frames over the catch season (October–April), were considered in our modeling analyses. The monthly models, utilizing more detailed data scales, exhibited the highest potential in identifying the overall distribution patterns of the fish, compared to temporally‐coarse BRT models. The best models were the BRTs fitted using data from March and April, which represented the final months of the catch season with the highest catch levels. In the monthly models, the main determinants of the Kutum's aggregation points were found to be dynamic variables including sea surface temperature, particulate organic and inorganic carbon, as opposed to static topographic parameters such as distance to river inlets. Seasonal and sub‐seasonal models identified particulate inorganic matter and distance to river inlets as the predictors with the highest influence on fish distribution. The geographical distributions of fish biomass hotspots revealed the presence of a stable number of fish aggregation hotspot points along the eastern coast, while some cold‐spot points were identified along the central and western coasts of the Caspian Sea. Our findings indicate that utilizing fine time scales in modeling analyses can result in a more reliable explanation and prediction of fish distribution dynamics. The investigated approach allows for the identification of intra‐seasonal fluctuations in environmental conditions, particularly dynamic parameters, and their relationship with fish aggregation.

## Graphical abstract

In our study, we attempted to investigate the key role of the temporal resolution of data in distribution modeling for an important fish species of the Caspian Sea, with high conservation and commercial importance. Our findings indicate that utilizing fine time scales in modeling analyses can result in a more reliable explanation and prediction of fish distribution dynamics. The investigated approach allows for the identification of intra‐seasonal fluctuations in environmental conditions, particularly dynamic parameters, and their relationship with fish aggregation.

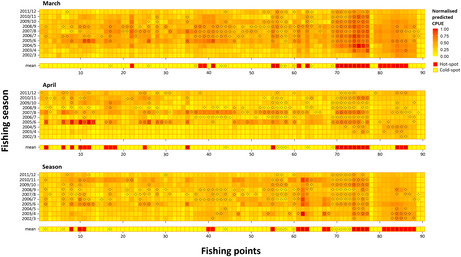

## 1 INTRODUCTION

Modeling and predicting the distribution and abundance of fish species is crucial for effective fisheries management (Li et al., 2016). Understanding the spatial distribution of fish species is essential for successful fisheries management, conservation planning, and assessing the impacts of environmental changes (Pennino et al., 2020). As a result, fish distribution modeling has received considerable attention (Giannoulaki et al., 2013; Guisan & Zimmermann, 2000).

The temporal resolution of data is an important factor in species distribution modeling analyses and can greatly influence the explanatory and predictive ability of models. Identifying appropriate distributional patterns of organisms requires determining the suitable temporal resolution of data (Fernandez et al., 2017; Scales et al., 2017), considering the ecological or management question (Mannocci et al., 2014, 2017), and spatiotemporal variability of environmental variables (Redfern et al., 2006). The temporal resolution of available environmental datasets is another important factor that typically affects the time frame of species modeling analyses (Jetz et al., 2012). Basic knowledge of the ecological characteristics of the species (e.g., migratory behavior) and variable dynamism (i.e., temporal variability) could be effective in selecting relevant environmental predictors and proper time resolutions (Fernandez et al., 2017). The limited studies that have examined the effects of temporal scale on the performance of distribution models suggested applying fine temporal resolutions to improve descriptive and predictive ability of models (Becker et al., 2016; Fernandez et al., 2017, 2018; Mannocci et al., 2017; Stelzenmüller et al., 2013).

Various computational techniques, including machine learning, have been employed to identify the habitat features that influence fish distributions (Elith et al., 2008; Froeschke & Froeschke, 2011; Hua et al., 2020; Li et al., 2015). Among the machine learning techniques, regression trees (RT) and boosted regression trees (BRT) modeling methods, which combine statistical and machine learning approaches, have been widely used to establish relationships between fish distribution patterns and environmental predictors (Anderson et al., 2016; Froeschke et al., 2010; Froeschke & Froeschke, 2011, 2016; Knudby et al., 2010; Leathwick et al., 2008; Pittman et al., 2007). More explanatory power and better predictive performance of this modeling technique have been reported compared to some commonly used statistical approaches, for example, the generalized additive model (GAM), which has extensive applications in ecological studies (Elith et al., 2006; Froeschke & Froeschke, 2016; Leathwick et al., 2006; Moisen et al., 2006).

Caspian Kutum (*Rutilus frisii*, Nordmann 1840), from the Leuciscidae family (Eagderi et al., 2022), is an endemic species of the Caspian Sea being distributed from the mouth of the Terek River in the north of the Caspian Sea, along the west and south coast to the mouth of the Atrak River (Rabazanov et al., 2019). Kutum with a life span of up to 9 years (average: 4.5 years) can reach a maximum length and weight of 58 cm and 3.5 kg, respectively (average length: 47.1 cm; average weight: 1.7 kg) (Khodorevskaya et al., 2014). Males and females mature at 2–3 and 3–4 years old, respectively (Khodorevskaya et al., 2014). Kutum has wintering and spawning migrations and as a migratory anadromous fish, it migrates into the rivers for spawning during March and April, with its peak in April (Afraei Bandpei et al., 2009). After spawning, the fish migrate back into the sea for feeding. The wintering migration of the fish takes place during December and January into deep waters (Afraei Bandpei et al., 2009).

Kutum is mainly an omnivorous fish. During the early stages of life history, it feeds on phyto‐ and zooplankton and insect larvae (Valipour et al., 2011), but the adult fish diets are mostly mollusks (especially bivalves), crustaceans, and polychaetes (Abdolmalaki et al., 2009; Khodorevskaya et al., 2014). The intensity of the feeding behavior of Kutum has temporal fluctuations, where the lowest feeding was reported during wintering and spawning migration periods (December to January, and April, respectively) (Afraei Bandpei et al., 2009).

In the Caspian Sea, sturgeons (especially in their fry stage) and fishes of the family Cyprinidae are the main species that feed on benthic organisms and may compete for prey items with the Caspian Kutum. However, considering the critical decreases in their abundance during the last decades (Fazli & Daryanabard, 2020; Karpinsky, 2010), it is not expected that this competition has had decreasing effects on the stocks of Kutum. Also, based on the conducted studies (Cites, 2017; Fazli et al., 2017; Tavakoli et al., 2019), population collapses of the main predators, including Caspian Seal (*Phoca caspica*) and sturgeon fishes (which can feed on young Kutum in their adult stage), have lowered predation pressure on Kutum's populations. Therefore, during the last two decades, nutritional competition and predation pressure have not been critical biotic factors influencing Kutum abundance.

Caspian Kutum has contributed nearly >70% of the total yearly catch of the bony fishes in Iranian waters (Esmaeili et al., 2015; Ghasemi et al., 2014) and thus has high commercial importance (Abdolhay et al., 2012). Kutum is mainly caught by small fishing cooperatives along the southern coastline of the Caspian Sea using beach seine. The catch season starts in September and lasts until April. The Iranian Fisheries Organization (IFO) conducted re‐stocking programs by releasing artificially‐produced fingerlings into the Sea (140–400 million fingerlings in 2000–2009; IFO, 2013). However, the catch has dropped from 1.9 × 10^4^ tons in 2000 to 1.6 × 10^4^ tons in 2014. Accordingly, the number of active fishing cooperatives decreased from >150 to <120 (IFO, 2013). Such decline stemmed from overfishing, water pollution, and degradation of riverine spawning areas (Ghani Nejhad et al., 2000; Rabazanov et al., 2019; Razavi Sayyad, 1999).

During the last three decades, fishing points of Kutum were in fixed locations over the Iranian coastal waters of the Caspian Sea, and it is not clear how the geographical positions of these fishing grounds were designated. Apparently, they are distributed randomly along the southern coastline of the Caspian Sea. Considering the overall decreasing trend in catch levels of the Kutum during the last decade and also, the consequent reduction in the number of active fisheries cooperatives, understanding the spatio‐temporal dynamics of the fish distribution patterns over southern Caspian Sea coastal waters and the roles of the relevant environmental variables in affecting its fluctuations is of high importance in planning effective conservation and management programs. In this regard, the present study aimed to: (1) model the spatiotemporal distribution of Kutum through the relationships between the fish catch and environmental parameters; (2) assess the effects of temporal resolution of the modeling analyses on descriptive and predictive performance of distribution models; (3) gain some knowledge about the effects of the most relevant environmental predictors and their temporal dynamics on fish distribution patterns; and (4) identify spatiotemporal patterns of fish distribution hot/cold‐spots occurrence.

## 2 MATERIALS AND METHODS

### 2.1 Fish catch data

**FIGURE 1:**
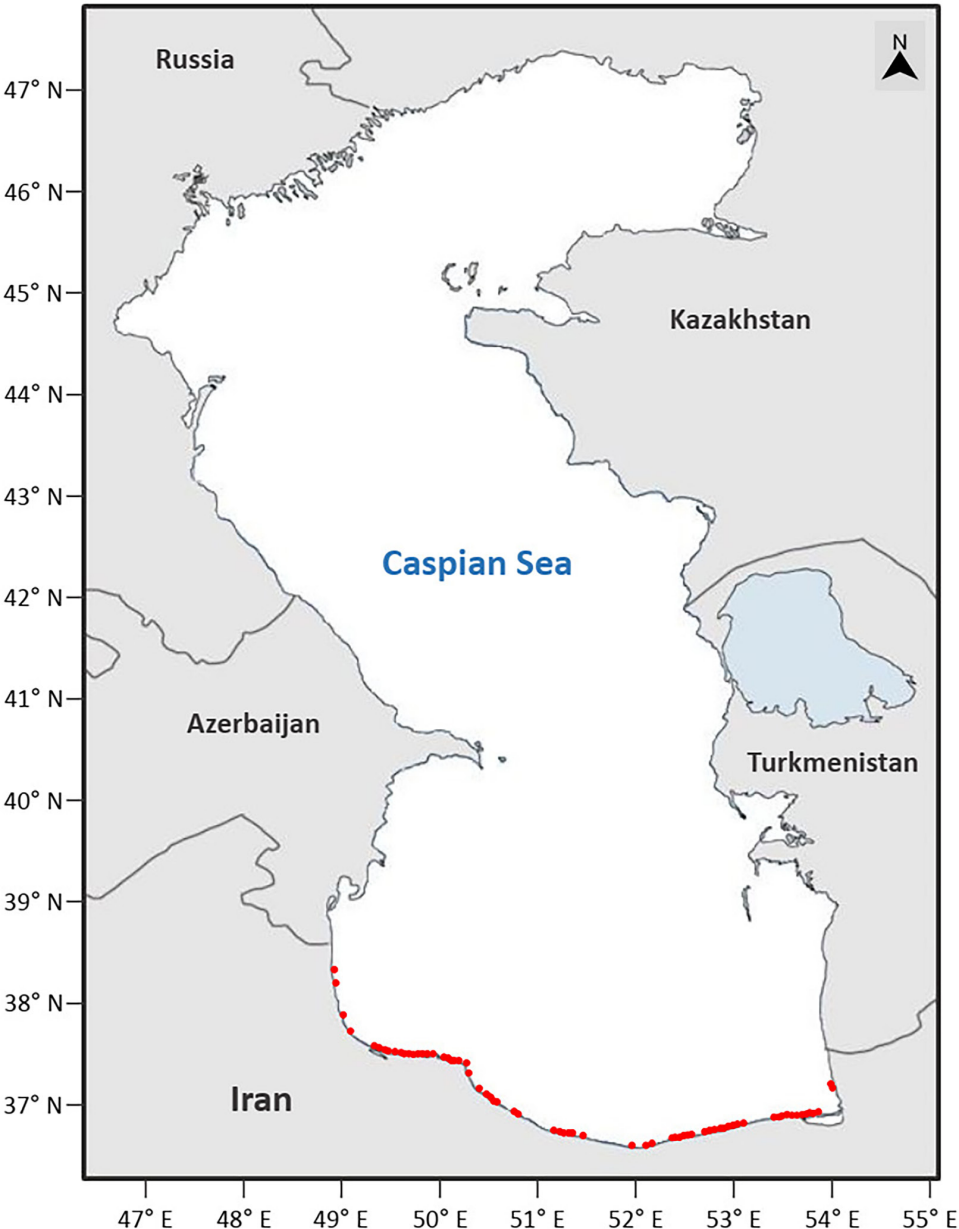
Geographical distribution of fishing cooperatives locations (•) for Caspian Kutum, *Rutilus frisii*, along the southern coast of the Caspian Sea.

**FIGURE 2:**
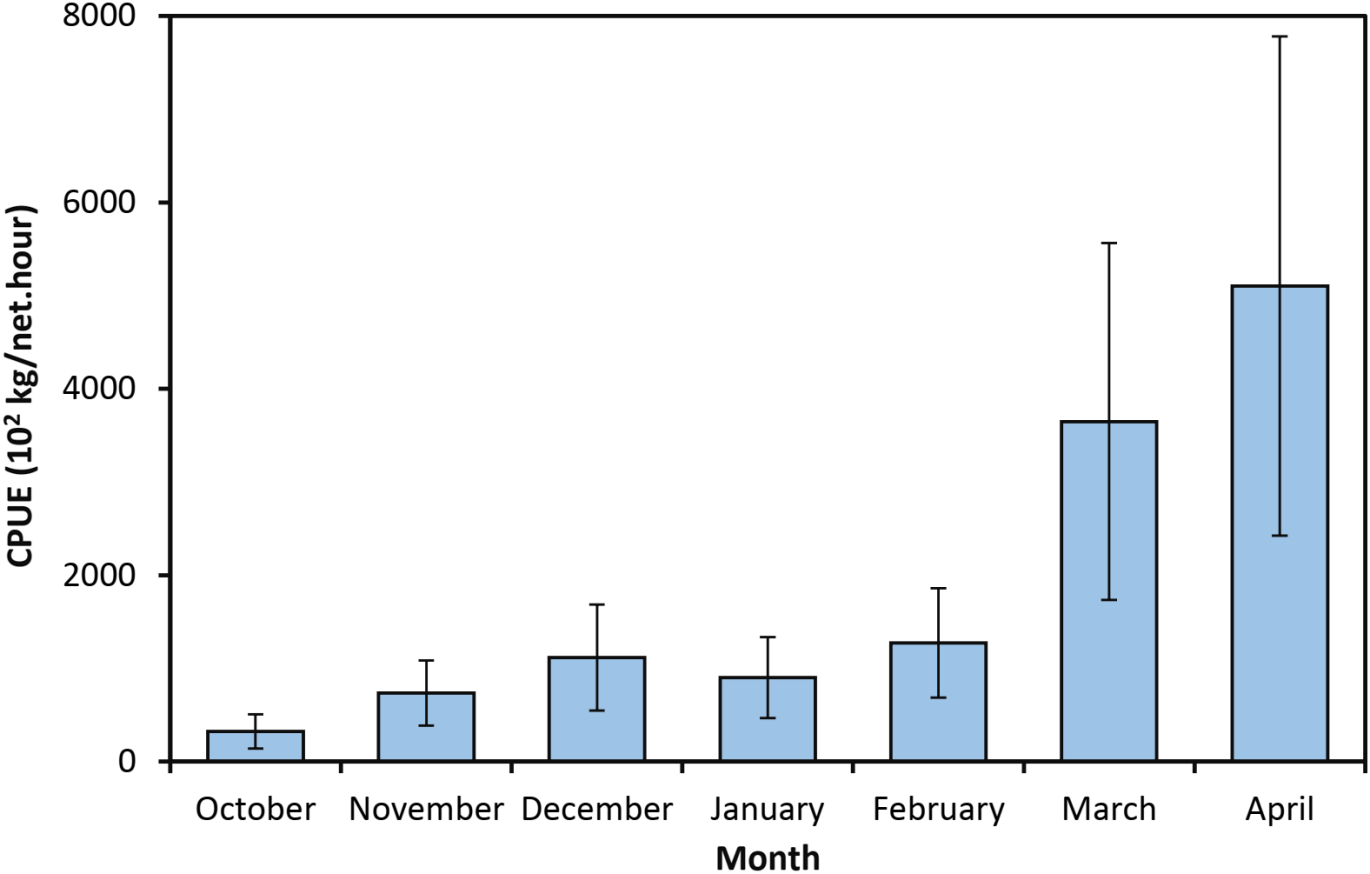
Overall monthly proportions of catch per unit of effort (CPUE) for Caspian Kutum, *Rutilus frisii*, during catch seasons (2002/3–2011/12).

The catch data of Kutum were obtained from the Iranian Fisheries Organization (IFO) for over 150 fishing points (cooperatives) along the Iranian coast of the Caspian Sea (Figure 1). The data included catch values (kg), fishing time (hours), and the number of dragged seine nets, covering the catch seasons from 2002/3 to 2011/12. The seine nets used had a length of 1200 m, a depth of 12 m, and mesh sizes of 33 and 45 mm at the bag and wings, respectively. Each fishing cooperative was assigned a specific geographical fishing zone. Out of the cooperatives, only 90 were consistently active throughout the study period, and their data were used for the modeling analysis. The catch season for the Caspian Kutum extends from September to April of the following year, with September excluded due to limited or no data available during that month. There was an increasing trend in catch levels over the catch season with a steep increase from February to March, when over 65% of the total seasonal catch occurred (Figure 2). Hence, three temporal frameworks were considered for modeling analyses: (1) monthly (the catch data were averaged over each month), (2) sub‐seasonal (the catch data were averaged over two periods: the early fishing period (EFP) from October to February and the last fishing period (LFP) from March to April), and (3) seasonal (the catch data were averaged over the whole catch season).

To standardize fish abundance, the catch levels were converted to catch per unit of effort (CPUE) using the following equation (Equation 1):

(1) $${\text{CPUE}}_{\mathrm{ijz}}=\frac{{\text{Catch}}_{\mathrm{ijz}}}{\text{Number of}\;{\text{seine nets}}_{\mathrm{ijz}}\times {\text{Fishing time}}_{\mathrm{ijz}}}$$

where CPUE_*ijz*_ is the CPUE (kg net^−1^ h^−1^) for the time period *i*, catch season *j*, and fishing location *z*.

### 2.2 Environmental data

Based on the prior studies, relevant remotely sensed environmental variables with reported potential ecological effects on fish distribution were selected as explanatory predictors to model CPUE distribution of Kutum over the fishing points (Griffiths et al., 2017; Guo et al., 2022; Ito et al., 2016; Mahowald et al., 2018; Moëzzi et al., 2022; Olsen, 2019; Parra et al., 2017; Pirtle et al., 2019; Stamoulis et al., 2018). The variables were the day‐time sea surface temperature (SST), near‐surface chlorophyll‐a concentration (CHL), aerosol optical thickness (ASL), particulate organic carbon (POC), particulate inorganic carbon (PIC), depth, slope, aspect, and distance to river inlets at fishing points (Table 1).

**TABLE 1:** Environmental variables used as predictors in the boosted regression trees models.

Variable	Unit	Resolution		Source/reference
		Spatial	Temporal	
Day‐time sea surface temperature (SST)	°C	4 km	Monthly (2002–2012)	MODIS (2021)^a^
Near surface chlorophyll‐*a* concentration (CHL)	mg m^−3^			
Aerosol optical thickness (ASL)	–			
Particulate organic carbon (POC)	mg m^−3^			
Particulate inorganic carbon (PIC)	mol m^−3^			
Depth	m	4 km	2021	GEBCO (2021)^b^
Slope	°	4 km	2021	Created using the bathymetric map
Aspect	°	4 km	2021	Created using the bathymetric map
Distance to the river inlets	km	4 km	2021	Local maps

^a^ MODIS: Moderate Resolution Imaging Spectroradiometer, United States National Aeronautics and Space Administration (NASA) Goddard Space Flight Center, Ocean Ecology Laboratory, 2021 (https://modis.gsfc.nasa.gov/).

^b^ GEBCO: General Bathymetric Chart of the Oceans, 2021 (http://www.gebco.net).

The depth was calculated as bathymetric data minus 27 as the water level difference between the Caspian Sea and open oceanic waters equals 27 m (Chen et al., 2017; Moëzzi et al., 2022). Slope and aspect maps were created from the bathymetry map. The distance of the fishing locations to the main water inlets was calculated as their nearest direct distance to the mouth of the main rivers along the coast. Data were converted to raster layers using the raster package (Hijmans, 2021) in R 4.1.2 (R Core Team, 2021).

The collinearity of predictor variables was assessed using Pearson correlation. Since all of the correlation coefficients were <0.7, there was no problematic collinearity among the predictors (Schickele et al., 2020), and all of them were used in fitting BRTs.

### 2.3 Model fitting and evaluation

Boosted regression trees (BRT) were used to investigate relationships between Kutum distribution and potential environmental predictors. BRT models explain response–predictor relationships using recursive binary splits and creating ensembles of regression trees (Elith et al., 2008). This modeling technique can handle missing values, capture non‐linear relationships, and minimize prediction errors when examining the correlations between predictor and response variables (Li et al., 2015). BRT model is applicable for continuous or categorical predictor variables and is not affected by data transformations or extreme observations (Froeschke & Froeschke, 2016). Moreover, this model can assess the relative importance of habitat predictors in relation to the response variable (Elith et al., 2008; Xue et al., 2017). BRT models were fitted using the gbm R package (Elith et al., 2008; Greenwell et al., 2020), with all environmental variables as predictors and CPUE as the response variable. Model parameters were automatically tested until obtaining the best fit using an interaction depth of 3, a learning rate of 0.01–0.001, a bag fraction of 0.75, and maximum trees of 10,000 with a Gaussian error distribution (Sievers et al., 2020). For each temporal scale, 80% of data (first eight catch seasons) were selected for model training, and the models were cross‐validated using the bootstrapping method (100 iterations; Kuhn & Johnson, 2013). Considering the probable tendency of BRT models to over‐fit training data, the remaining 20% of data (i.e., the last two catch seasons) were used as a completely unknown dataset for testing of the models (Li et al., 2017). The goodness‐of‐fit of the models was assessed using adjusted *R*‐squared (adj‐*R* ^2^). The models fitted for each temporal scale were examined using the testing datasets of all temporal scales, and their predictive performance was evaluated using normalized root mean squared error (nRMSE) scores, calculated as follows:

(2) $$\text{nRMSE}=\frac{\sqrt{\frac{\sum_{i=1}^{N}{\left(x_{i}-{\hat{x}}_{i}\right)}^{2}}{N}}}{\left(x_{\max}-x_{\min}\right)}$$

where *x*_*i*_ is the raw value, ${\hat{x}}_{i}$ is the predicted value, *N* is the number of observations, and *x*_max_ and *x*_min_ are the maximum and minimum scores of the raw data, respectively. Also, correlation coefficients (*r*) of fitted linear models between raw and fitted values of the models (for both training and testing data) were calculated for examining the accuracy of the model's predictions.

The relative importance (RI) scores of predictor variables were calculated using the VarImp function of the caret package in R (Kuhn & Johnson, 2013). Since there were nine predictors and the total values of RIs should equal 100%, variables with mean RI scores greater than 11.11% (=100/9) were considered as influencing predictors (Thorn et al., 2016). The difference in mean RIs of each predictor from this significance level (11.11%) was statistically assessed using a one‐sample *t*‐test (*α* = 0.05). Partial dependency plots of significant variables were used to examine fluctuations in CPUE over the ranges of the predictors.

### 2.4 Fish hot/cold‐spot determination

A 0.8‐quantile of the raw and estimated CPUE values was used as the threshold to determine fish distribution hot‐ and cold‐spots (Li et al., 2016), where a hot‐spot was defined as a fishing point with CPUE ≥0.8‐quantile and the rest of the points as cold‐spot locations. The spatiotemporal accuracy of BRT models in predicting the occurrence of hot/cold‐spots was evaluated and compared between different temporal scales by overlaying observed and predicted CPUEs.

## 3 RESULTS

### 3.1 Model performance

Fitting BRT models to training datasets with different temporal resolutions showed that monthly models had generally higher explanatory power compared to sub‐seasonal (EFP and LFP) and seasonal models (Table 2). Among the monthly models, the best fitted models were obtained for October, March, and April (having higher adj‐*R* ^2^). These models showed higher correlation coefficients (*r*_Train_) between fitted and observed values of training datasets (Table 2). The models of the other months showed similar weak fits. However, predicting testing data using fitted models demonstrated that March and April BRTs with the lowest nRMSE scores (0.49 ± 0.19 and 0.35 ± 0.12, respectively) had the best predictive performance (Table 3). Predictions of other monthly models presented lower predictability compared to the sub‐seasonal and seasonal BRTs. Also, predictions of March and April BRTs for testing datasets of other temporal scales generally led to more accurate estimates (with lower nRMSE and higher *r*_Test_ values) compared to the predictions obtained for other BRTs with non‐identical testing datasets (Tables 2 and 3). Accordingly, BRT models of March and April were selected as the models with the best performance.

**TABLE 2:** Boosted regression trees (BRT) models performance measures (mean ± standard deviation) averaged over 100 bootstrapped iterations.

Model		Adj.*R* ^2^	*r*_Train_	*r*_Test_
Monthly	October	0.358 ± 0.033	0.59 ± 0.11	0.09 ± 0.05
	November	0.167 ± 0.063	0.41 ± 0.08	0.27 ± 0.12
	December	0.143 ± 0.091	0.37 ± 0.06	0.04 ± 0.03
	January	0.158 ± 0.084	0.41 ± 0.07	0.02 ± 0.02
	February	0.205 ± 0.111	0.45 ± 0.12	0.23 ± 0.11
	March	**0.324** ± **0.072**	**0.57** ± **0.09**	**0.49** ± **0.19**
	April	**0.288** ± **0.095**	**0.43** ± **0.06**	**0.35** ± **0.12**
Sub‐seasonal	EFP	0.148 ± 0.043	0.41 ± 0.08	0.08 ± 0.04
	LFP	0.185 ± 0.019	0.42 ± 0.07	0.22 ± 0.08
Seasonal		0.112 ± 0.037	0.33 ± 0.09	0.19 ± 0.10

*Note*: Bold rows show months with models selected for further analysis.

Abbreviations: Adj.*R* ^2^, Adjusted *R* ^2^; EFP, Early fishing period; LFP, Last fishing period; *r*_Train_, Correlation coefficient between raw and estimated values for training data; *r*_Test_, Correlation coefficient between raw and estimated values for testing data.

**TABLE 3:** Averaged normalized root mean squared error (nRMSE) scores for predicting all testing datasets by fitted boosted regression trees (BRT) models.

		Testing data									
		Oct	Nov	Dec	Jan	Feb	Mar	Apr	EFP	LFP	Season
Model	Oct	1.157	1.113	3.328	2.913	2.891	4.896	8.166	3.274	8.782	3.579
	Nov	1.344	1.132	1.830	1.736	1.733	2.614	4.376	1.871	5.013	2.343
	Dec	1.235	1.008	1.192	1.745	1.463	1.985	2.917	1.264	3.023	1.533
	Jan	1.078	0.993	1.129	1.127	1.130	1.468	1.625	1.067	1.875	1.095
	Feb	1.269	1.136	1.033	1.351	1.043	1.211	1.231	1.001	1.812	1.035
	Mar	1.366	1.277	1.141	1.229	1.212	0.927	1.078	1.208	1.003	1.162
	Apr	1.583	1.510	1.288	1.430	1.305	1.080	0.984	1.322	1.032	1.284
	EFP	1.461	1.132	1.587	1.801	2.065	2.861	4.695	1.132	5.029	2.164
	LFP	1.568	1.484	1.288	1.363	1.241	1.053	1.059	1.364	1.079	1.312
	Season	1.615	1.399	1.008	1.301	1.079	1.123	1.811	1.158	1.920	1.118

*Note*: Shaded cells indicate the lowest nRMSE scores.

Abbreviations: EFP, Early fishing period; LFP, Last fishing period.

### 3.2 Environmental predictors

**FIGURE 3:**
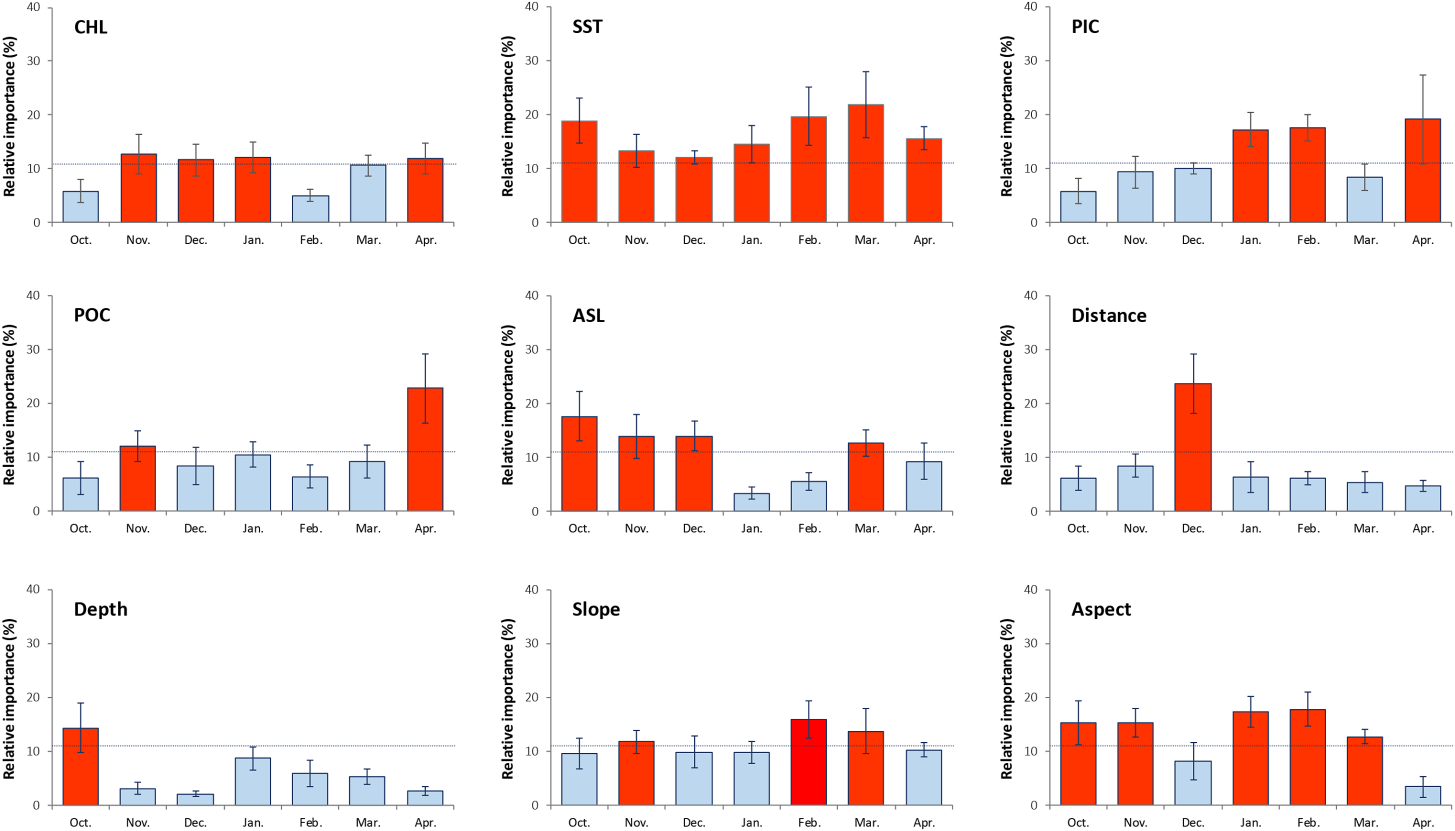
Mean (±SD) relative importance (RI [%]) of environmental predictors in monthly models. The horizontal dotted line shows the significance level of relative importance scores (11.11%). The vertical bars in red indicate a significant RI score for the variables. ASL, Aerosol optical thickness; CHL, Near surface chlorophyll‐*a* concentration; Distance, Distance to the river inlets; PIC, Particulate inorganic carbon; POC, Particulate organic carbon; SST, Day‐time sea surface temperature.

**FIGURE 4:**
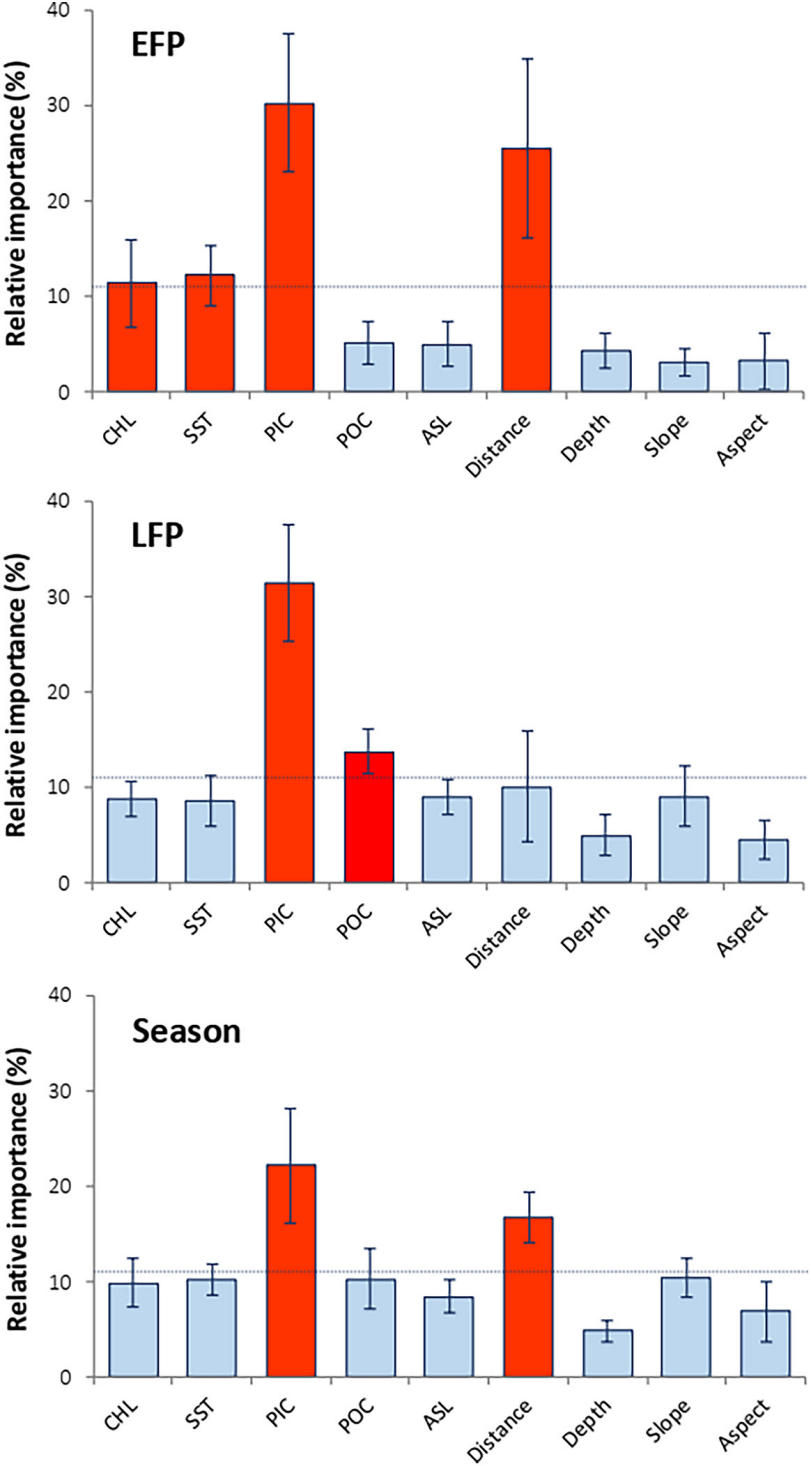
Mean (± standard deviation) relative importance (RI [%]) of environmental predictors in sub‐seasonal (EFP, Early fishing period; and LFP, Last fishing period) and seasonal models. The horizontal dotted line shows the significance level of relative influence scores (11.11%). The vertical bars in red indicate a significant RI score for the variables. ASL, Aerosol optical thickness; CHL, Near surface chlorophyll‐*a* concentration; Distance, Distance to the river inlets; PIC, Particulate inorganic carbon; POC, Particulate organic carbon; SST, Day‐time sea surface temperature.

Different environmental predictors were identified as the main drivers of Kutum distribution in the fitted BRTs. Among all predictors, SST had a significant influence in all monthly models, with the highest RI scores observed in October, February, and March (Figure 3), as well as in the EFP (Figure 4). Particulate inorganic carbon (PIC) had considerable effects on Kutum CPUEs in the EFP, LFP, and seasonal models (Figure 4). Additionally, PIC was found to be an influential predictor in January, February, and March. The RI scores of POC were significant for BRT models of November, April, and LFP. Aerosol optical thickness (ASL) was an effective predictor in models from October to December and March. CHL (Chlorophyll‐a concentration) showed low but significant RI scores in BRTs of November to January, April, and also in the EFP. Among the topographical parameters, the RI score for distance from the river mouth was significant in December, EFP, and seasonal BRT models. Other topographic parameters such as depth, slope, and aspect influenced fish CPUEs only in one or some of the monthly models.

**FIGURE 5:**
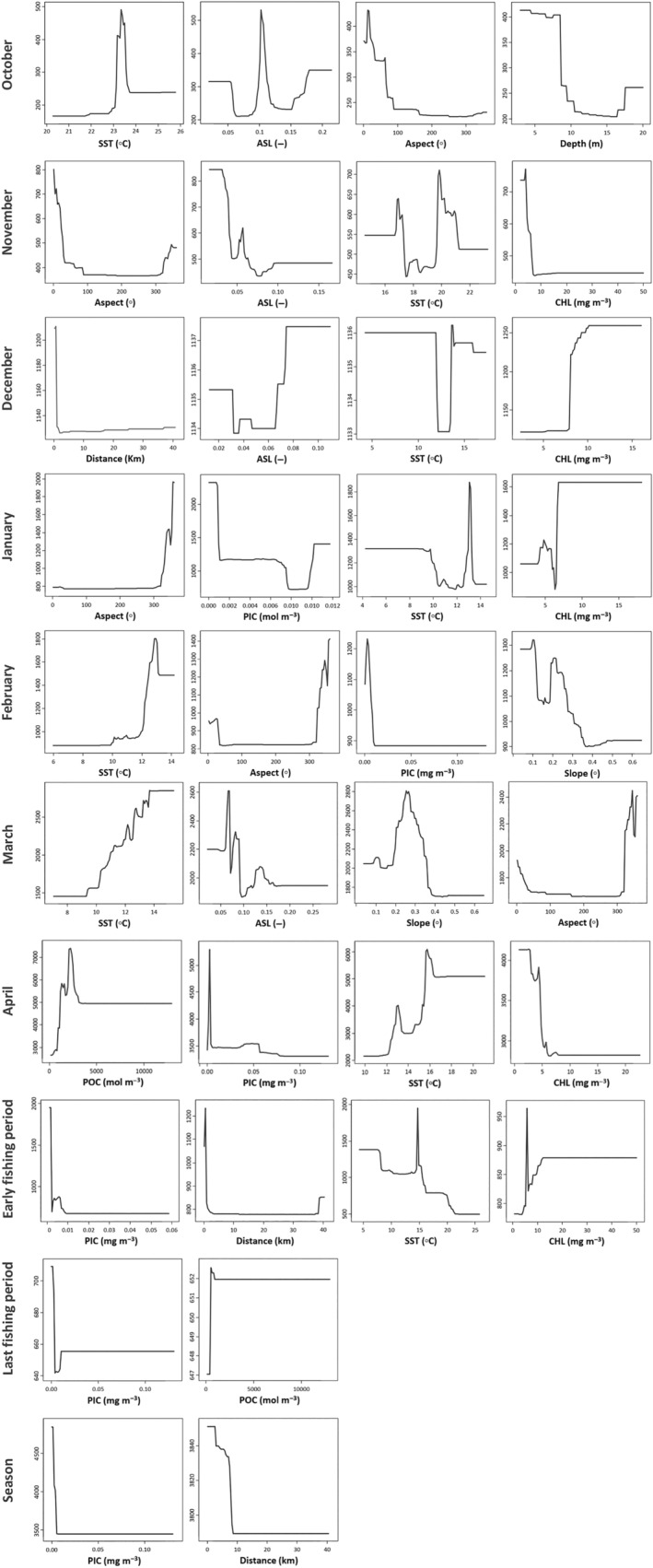
Mean partial effect plots of the environmental predictors with significant influence on catch per unit of effort (CPUE) for monthly, sub‐seasonal (early fishing period and last fishing period), and seasonal boosted regression trees (BRT) models. ASL, Aerosol optical thickness; CHL, Near surface chlorophyll‐*a* concentration; Distance, Distance to the river inlets; PIC, Particulate inorganic carbon; POC, Particulate organic carbon; SST, Day‐time sea surface temperature.

The relationships between CPUE fluctuations and environmental predictors were assessed using partial dependence plots (Figure 5). SST had a clear increasing trend over the period of February to April with maximum effects at higher temperatures, while there was a decreasing trend for EFP. In all BRT models with PIC as a significant predictor, its highest partial effects were found at the range from 0.00 to 0.01 mol m^−3^. There were increasing trends for POC effects with the highest influences being found from 2000 to 4000 mg m^−3^ in April and for concentrations greater than 1000 mg m^−3^ in LFP. The ranges of CHL and ASL with the highest partial effect were different between models. The maximum effects of distance from river inlets were found in a distance range lower than 2 km in December and EFP models, while for the seasonal model, the highest effects belonged to a more extended distance range (0–10 km). The fishing points with depths less than 10 m, slopes between 0.2–0.4°, and aspect <40°and >300° were areas with the highest partial effects of these parameters obtained from the BRTs with the significant RIs of these variables.

### 3.3 The distribution of the hot−/coldspot points

**FIGURE 6:**
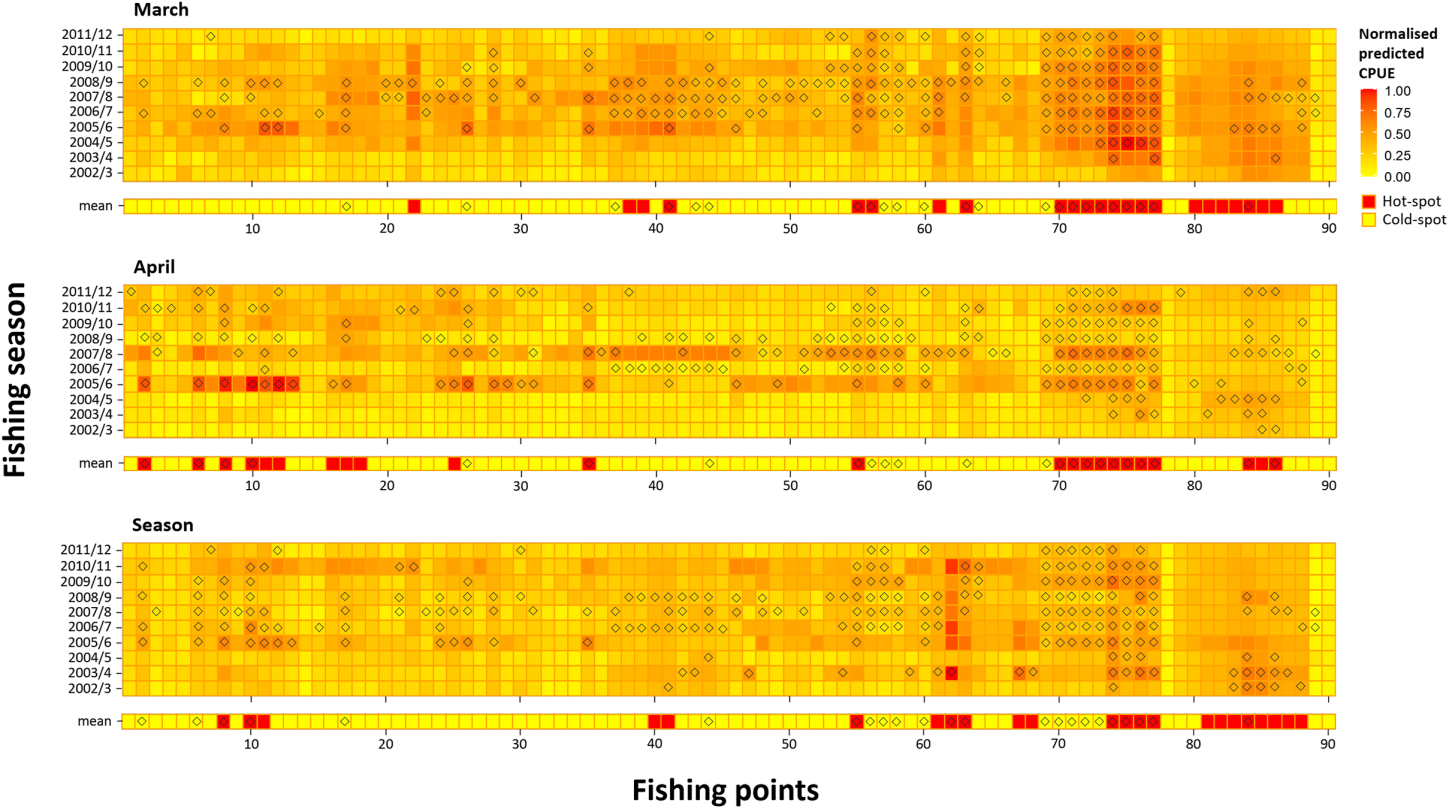
Visualization of normalized predicted catch per unit of effort (CPUE) of March, April, and seasonal boosted regression trees (BRT) models for catch seasons 2002/3 to 2011/12. The (⬨) symbol indicates a hotspot point, as a fishing point with CPUEs >0.8‐quantile of observed CPUE data, over each temporal scale.

**FIGURE 7:**
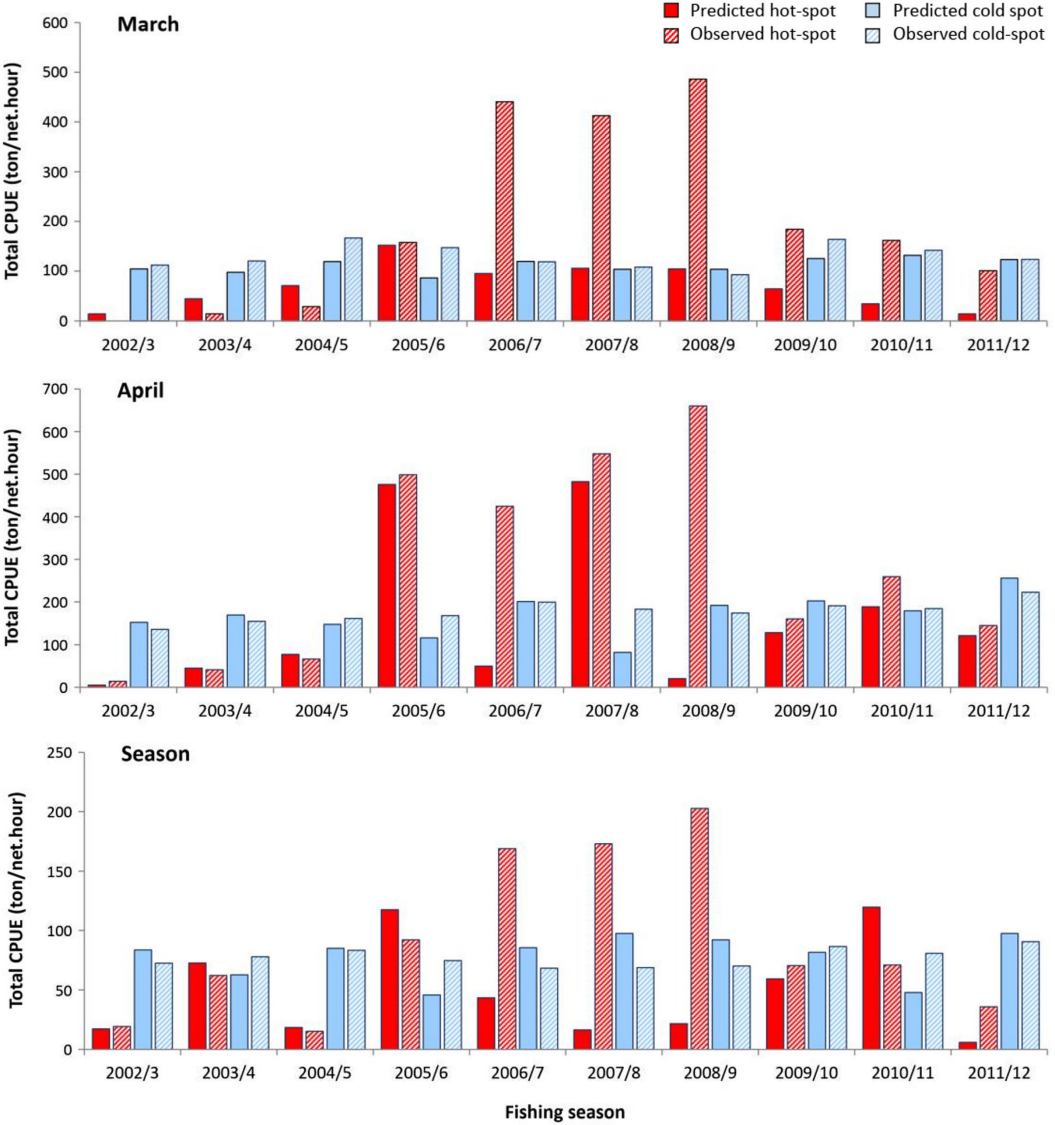
Total catch per unit of effort (CPUE) (ton/net. hour) in predicted and observed hot−/coldspot points for the monthly (March and April) and seasonal models during catch seasons of 2002/3 to 2011/2012.

Comparing the spatial incidence of observed and predicted hot−/coldspots of Kutum distribution showed that BRT models of March and April had higher accuracy in identifying hot−/coldspots than the seasonal model in each and all catch seasons (Figure 6). The highest proportion of hotspot locations was found in March. There were higher differences between predicted and observed total CPUEs of hot−/coldspots for the seasonal model than those of the March and April BRTs (Figure 7), except for catch seasons of 2005/6 to 2008/9, where higher differences existed between predicted and real CPUEs for all models.

Spatial distributions of fish hotspot locations for monthly (March and April) and seasonal models (Figure 6) indicated that the hotspot points of Kutum were mainly located in the eastern coast of the southern Caspian Sea. There was a considerable number of fish hotspots in April during catch seasons 2005/6 to 2008/9 in the central and western regions of the coastal line, which were not identified using the seasonal model outputs.

**FIGURE 8:**
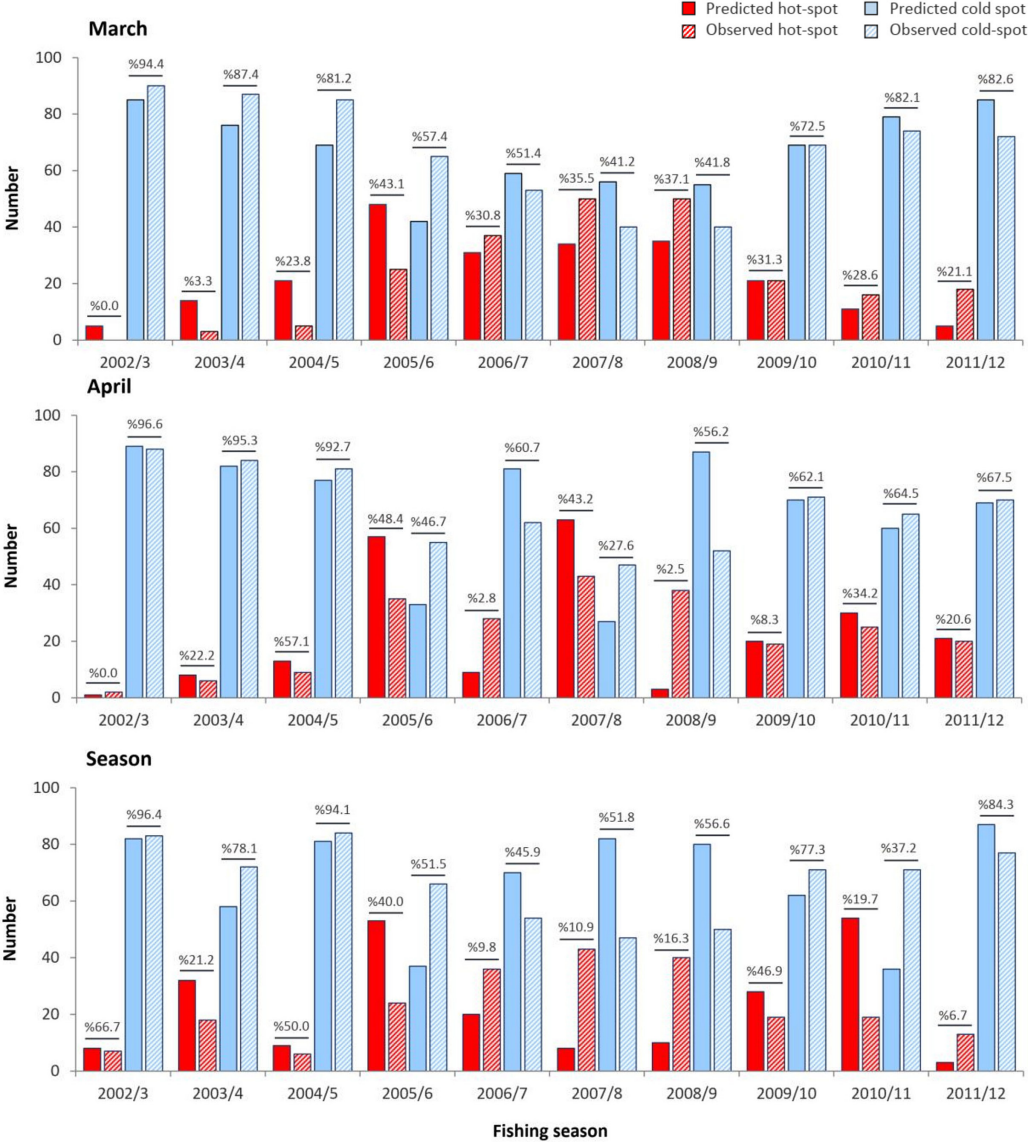
Predicted and observed hot−/coldspot point proportions of landing points for the monthly (March and April) and seasonal models during catch seasons of 2002/3 to 2011/2012. The values on the bars show the overlap (%) of predicted and observed hotspot and coldspot points in each catch season.

Based on the March and April models, the proportions of the hotspot points increased from zero in the catch season of 2002/3 to the highest number in 2005/6–2008/9, and then decreased until the last catch season (Figure 8). The rate of such increase was higher in March than in April. In contrast, the outputs of the seasonal BRT showed irregular changes in the hot−/coldspot proportions of fishing points during the catch seasons. The observed and predicted hot−/coldspot points overlapped at a higher rate in the selected monthly models than those of the seasonal models.

## 4 DISCUSSION

The Caspian Sea, the largest enclosed body of water on Earth, is home to a diverse range of fish species. Understanding the distribution patterns of these species is of great importance for effective fishery management and conservation efforts. In the present study, we aimed to investigate the spatiotemporal distribution dynamics of Caspian Kutum (*Rutilus frisii*) in the Caspian Sea emphasizing the influence of data temporal resolution on the distribution modeling accuracy.

The choice of temporal resolution in ecological modeling is a critical consideration, and the present study that focused on the Caspian Kutum distribution modeling has demonstrated that temporally fine‐resolution data, specifically on a monthly basis, yield the most robust and informative models. The best describing and predictive performance of models were obtained with BRTs fitted to the finest‐scaled data (i.e., monthly models) but with considerably higher proportions of catch (i.e., March and April). Also, according to the differences in estimations for total CPUEs than the observed values (Figure 6), and trends in predicted and observed hotspot proportions and their spatial overlap (Figures 7 and 8), it was clear that monthly models had better performance and higher accuracy than the seasonal model. Although using data with a smaller time scale has led to the best estimates of the species distribution, but this situation has only been found for time intervals when the fish had a non‐random distribution over fishing points. This finding may suggest that Kutum occurs sporadically and unpredictably along the Caspian Sea coast from summer to winter. However, it is abundant in spring almost in every place of the Sea. It has been suggested to use appropriate temporal partitioning in species distribution modeling, considering the ecological characteristics of the species and the environmental variability of the ecosystem (Mannocci et al., 2017; Roberts et al., 2016). The importance of incorporating the season in studying the distribution of Caspian Kutum has already been used (Fazli et al., 2010; Valipour et al., 2011), and it has been reported that Kutum is sparsely distributed over shallow coastal waters during winter (Afraei Bandpei et al., 2009). The models that used low winter catch data as fish abundance measure (directly in monthly BRTs of October to February and indirectly in EFP and seasonal BRTs), as well as the unrelated or weakly related environmental covariates for these time frames, entered outlying data into the modeling process and consequently led to inconsistent predictions of fish distribution patterns. Thus, it may be necessary to use relevant and desired temporal slices of data (e.g., species abundance and environmental variables) to obtain reliable distribution models.

The dynamism of the environmental predictors is one of the main factors in studying the effects of temporal resolution of data on the performance of species distribution models, where the effects of highly dynamic variables can be revealed only in finer temporal resolutions (Fernandez et al., 2017). Such factors are of greater importance for organisms having movement behaviors dependent on the changes in environmental conditions (e.g., feeding or breeding migrations) (Mannocci et al., 2017); in other words, the distribution patterns of species like Kutum with migratory behavior that takes place over yearly periods, can be explained more based on the spatiotemporal changes in dynamic parameters rather than being a function of static parameters such as topographical variables. In general, dynamic variables (e.g., SST, CHL, ASL, PIC, and POC) had larger contributions in monthly models, especially for March and April (with the highest catch levels), which was also evident for LFP. However, most topographic parameters (as static variables) had lower significance in defining fish distribution in these models. On the other hand, there were only PIC and distance from the river inlets, as the main predictors in the seasonal models, both of them with a distinct limited range of high effects. In these models, averaging the data over the entire fishing seasons led to hiding the fluctuations and variances of the dynamic parameters; therefore, only the factors with a limited effective range related to the fish abundance, especially for PIC (which in most models had similar marginal effect trends), were determined as the main factors affecting the distribution of fish. Consequently, the failure to recognize strong relationships between fish abundance and the real influencing environmental variables resulted in less accurate and unreliable model predictions (Fernandez et al., 2017). Therefore, using finer resolution of data could help us in finding relevant species‐environment relationships and obtaining distribution models with much better performances.

The findings from monthly models as well as monthly catch proportions largely reflect the life history of Kutum. From October to January, as a result of the decline in water temperature and formation of thermal stratification, the fish migrates into deep offshore waters for wintering leading to decreases in its abundance over shallow coastal regions. In all of the monthly models, SST was one of the main parameters affecting fish distribution, while for the models with lower temporal resolution, this variable was only significant in EFP at low contributions. The sea‐surface temperature was the first and the third important predictor of Kutum abundance in March and April, respectively, where higher abundances of fish were observed with an increase in temperature, while in the EFP model, an irrelevant decreasing trend of CPUEs was observed over the SST range. The increase in occurrence over high SSTs indicated that Kutum preferred high temperatures in the range of 8–20°C. In fact, the highest abundance coincided with temperatures of 16–20°C in April. Temperature, as an effective environmental factor in relation to fish distribution, controls survival and growth (Hua et al., 2020; Kempf et al., 2013; Olsen, 2019; Youcef et al., 2013), and winter migration of Kutum to deep offshore waters (Fazli et al., 2010; Valipour et al., 2011). Moreover, an increase in the temperature in coastal waters is indirectly coincided with high nutrient levels and hence availability of the prey (Pang et al., 2015). However, the water temperature has seldom been reported as an insignificant factor in the distribution of this species (Vayghan et al., 2013). Such contradiction may be related to the higher effect of this parameter in shallow coastal waters compared to deep offshore regions reported by Vayghan et al. (2013).

Our findings significantly depicted a clear temporal shift in main predictors of Kutum distribution across March and April (i.e., months with the highest catch levels), from mainly physical (SST, aspect, slope, and ASL) to mostly nutritional variables (POC, PIC, and SST). The LFP model also showed this situation with only POC and PIC as factors determining Kutum distribution. In April, when water temperature increased over most fishing locations, nutritional factors like POC and PIC became more important in providing the optimal habitat range for Kutum. This fish starts its migration to the coastal waters for feeding with increasing water temperature during this period. These two factors reflect the productivity of the water environment (Griffiths et al., 2017; Groom & Holligan, 1987; Kutti et al., 2008; Perea‐Blazquez et al., 2012; Rost & Riebesell, 2004). Higher POC and PIC levels are related to higher fluxes of carbon to the sea floor from overlying water column (Groom & Holligan, 1987; Kutti et al., 2008; Rost & Riebesell, 2004) which can lead to improving secondary production of benthic invertebrates (Griffiths et al., 2017) especially in coastal regions (Perea‐Blazquez et al., 2012), which are the main food elements for Kutum. An increasing trend of fish biomass was found over POC of 0–3500 mg m^−3^. However, the narrow optimum range of PIC (<0.01 mol m^−3^) preferred by the fish could be related to the negative effect of PIC on light penetration into the water body, since its high concentrations can cause between 10% and 90% light backscattering in marine systems (Balch et al., 1991, 1999). Accordingly, we can suggest that the distribution of Kutum in the last months of the catch season is firstly dependent on water temperature elevation, and after that, the incidence of favorable feeding grounds could be identified with their high POC contents.

Considering the reproductive migration of Kutum, which takes place over March to April, the highest densities of fish must have been observed in fishing points near the mouths of the main rivers entering the southern Caspian Sea. However, distance from the river inlet was not among the significant parameters of March and April, nor the LFP BRTs. Due to the lack of data about the biological characteristics of the catch (e.g., length, weight, and reproductive status of fish) in our dataset, it is not possible to explain this situation accurately. However, without a relevant relationship between distance and fish abundance during the period of fish reproductive migration, it can be said using sein nets with stable characteristics (i.e., mesh size) for more than one decade has led to declines in fish size and age at maturity with harvested fishes mainly having a size range larger than a fixed threshold, which has been reported in some studies that performed biological analysis of Kutum catch over limited coastal extents (Afraei Bandpei et al., 2009; Fazli et al., 2010). Therefore, in our modeling analyses, the importance of the reproductive migration of fish on its distribution was less inferable compared to its migration into the proper coastal feeding grounds, over the studied time period.

In the present study, the biotic relationships of the Kutum with its predator and prey species were not considered in the modeling process due to the lack of data for the studied decadal period and over the broad geographical extent of the fishing points. However, incorporating such data in the distribution modeling of this species could lead to models with much higher explaining and predictive power. Therefore, using these predictors in modeling analyses is suggested for future research works. Also, based on some of the conducted studies, it has been proposed to consider the lagged‐time effects of environmental parameters, especially SST and chlorophyll concentration, in the distribution modeling of marine organisms at higher trophic levels (Olden & Neff, 2001; Trujillo & Thurman, 2016; Wang et al., 2018). In our research, due to the use and comparison of different temporal frameworks of data in modeling analyses, it was not possible to apply such lagged effects for the seasonal and sub‐seasonal datasets; however, the use of such terms in monthly models can improve recognizing the temporal trends of the influencing levels of these variables on fish distribution.

Geographical distribution of fish hot−/coldspots obtained from the monthly BRT models mainly showed multiple hot‐spot incidence ranges over the western, central and eastern parts of the southern Caspian Sea coast. However, the temporal stability of hotspot occurrence over the eastern coasts was higher which was observed from the monthly (March and April) and seasonal models, while fish biomass hotspots over the central and western coastal regions which were obtained from the March model, were only observed during catch seasons 2005/6 to 2008/9. This temporal pattern in spatial occurrence indicated higher stability of environmental and ecological habitat conditions in the eastern coastal ranges preferred by Kutum. Vayghan et al. (2013) reported a spatial pattern of suitable habitat distribution with the highest suitability for Kutum in offshore waters in central and eastern coastal regions that partly supported our results. Considering these distributions, it could be said that, using monthly models, we can identify intra‐seasonal fluctuations in fish abundance hotspots, which were not obtained from the seasonal model.

## 5 CONCLUSION

In the present research, we attempted to understand the spatiotemporal dynamics of Caspian Kutum distribution over the southern coastal waters of the Caspian Sea, emphasizing the effect of temporal resolution of data on modeling performance and predictions. The combined use of the different temporal resolutions of data from different sources and boosted regression trees (BRT) modeling technique, considering the general temporal patterns of fish catch and ecological characteristics of the studied species with migratory behavior, led to the recognition of some key points related to its spatiotemporal dynamism. Our findings indicated that using finer time scales in modeling analyses could lead to more reliable explanations and predictions of fish distribution dynamics by identifying the intra‐seasonal fluctuations of environmental conditions, especially for the dynamic parameters, and their relations with fish aggregation. Based on the results, incorporating the data of time periods with low catch levels in the averaged dataset with coarser temporal resolution can musk the real patterns and dynamism of habitat parameters and consequently fish distribution. For the Kutum, with considerably much higher catch levels during the last months of the catch season, we found that despite the reproductive migration of the fish, water temperature and nutritional factors (e.g., POC) were the main detrimental drivers of fish hotspot delineation, which could only be detected using monthly BRT models. Also, predictions obtained from the monthly models obviously showed the key role of the temporal framework of data in determining the intra‐ and inter‐seasonal spatial changes in fish hotspot incidence over the decadal study period. The obtained results of this study could practically help Iranian fisheries managers to adopt more appropriate management policies regarding Kutum fisheries with special attention to the spatiotemporal distribution dynamics.

## AUTHOR CONTRIBUTIONS

**Fateh Moëzzi:** Conceptualization (equal); data curation (equal); formal analysis (equal); methodology (equal); software (equal); writing – original draft (equal). **Hadi Poorbagher:** Conceptualization (equal); formal analysis (equal); methodology (equal); supervision (equal). **Soheil Eagderi:** Conceptualization (equal). **Jahangir Feghhi:** Conceptualization (equal); methodology (equal). **Carsten F. Dormann:** Conceptualization (equal); methodology (equal). **Sabah Khorshidi Nergi:** Data curation (equal). **Kaveh Amiri:** Data curation (equal).

## CONFLICT OF INTEREST STATEMENT

The authors of this article declare that there is no conflict of interest.

## Data Availability

All data used in this research are publicly available (sources provided in the Methods). The fish catch data were available from the Iranian Fisheries Organization.
